# ARID4B: An Orchestrator from Stem Cell Fate to Carcinogenesis

**DOI:** 10.3390/cells14120872

**Published:** 2025-06-10

**Authors:** Rakhee Rathnam Kalari Kandy, Madan Kumar Arumugam, Mukesh Pratap Yadav, Bibhuti Bhusan Mishra, Jyotika Sharma

**Affiliations:** 1 Department of Critical Care Medicine, University of Texas MD Anderson Cancer Center, Houston, TX 77030, USA; rkalari@mdanderson.org; 2 Cancer Biology Lab, Centre for Molecular and Nanomedical Sciences, Sathyabama Institute of Science and Technology, Chennai 600119, Tamil Nadu, India; madankumar@sathyabama.ac.in; 3 Marlene and Stewart Greenebaum Comprehensive Cancer Center, School of Medicine, University of Maryland, Baltimore, MD 21201, USA; myadav@som.umaryland.edu; 4 Department of Developmental Dentistry, University of Texas San Antonio, San Antonio, TX 78229, USA; mishra@uthscsa.edu

**Keywords:** ARID4B, epigenetics, mESC, miRNA, AT rich interacting domain, spermatogenesis, hematopoiesis

## Abstract

All biological processes, from embryonic development to cancer, are tightly controlled by the interactions between genetics and epigenetics. An array of epigenetic modifications, such as DNA methylation, histone/chromatin modifications, and noncoding RNA-mediated targeting, are essential to regulate the heritable changes that occur during multiple cellular processes. A failure in proper regulation results in inappropriate gene expression that ultimately leads to pathological states. Groundbreaking advances in genomics and transcriptomics have revealed the potential involvement of epigenetics in various physiological and pathological states. The promising clinical and preclinical results shown by epigenetics drugs further underscore the central role of epigenetics in multiple human diseases, including cancer. AT rich interaction domain (ARID)-containing proteins are a family of evolutionarily conserved DNA binding proteins that regulate epigenetic modifications. Genome sequencing has revealed the existence of 15 ARID family proteins that are divided into 7 subfamilies based on their sequence and domain homology. Although the ARID family of proteins are implicated in cell growth, development, differentiation, and cancer, the diverse biological functions of many family members remain to be elucidated. Here, we focus on ARID4B to summarize its prominent role in embryonic stem cell differentiation and human malignancies.

## 1. Introduction

### 1.1. Epigenetic Regulation of Cell Fate/Cellular Phenotype

An explosion of knowledge in the field of genomic and proteomic technologies in the past several decades has unraveled the complexity and plasticity of epigenetics. The term epigenetics, coined by Conrad Waddington in 1942 [1], describes the heritable changes that occur in the cellular phenotype independent of any alterations in the primary DNA sequences. Epigenetic modifications play a cardinal role in the regulation of DNA-based processes that include replication, repair and transcription [2]. Fine-tuned epigenetic modifications, such as DNA methylation, histone/chromatin modifications, and noncoding RNA-mediated targeting, are essential to regulate the heritable changes that occur during multiple cellular processes. The failure of such regulation could result in improper gene expression that ultimately leads to pathological states [3,4]. The promising clinical and preclinical results shown by epigenetics modulating drugs underscore the central role of epigenetics in multiple human diseases, including cancer.

### 1.2. AT Rich Interaction Domains-Containing Protein Family

AT rich interaction domain (ARID)-containing proteins belong to a family of evolutionarily conserved DNA binding proteins found in eukaryotic kingdoms [5]. In search of AT-rich DNA biding proteins, the ARID domain was initially identified in the murine B cell-specific transactivator of IgH transcription (Bright) and Drosophila Dead Ringer (Dri) [6,7]. Based on the shared features of Bright and Dri proteins, this novel DNA binding domain was identified as ARID, reflecting its binding preference to AT-rich sequences [8]. ARID domain-containing proteins have been identified from numerous species, including insects, nematodes, yeasts, plants, and mammals [9]. ARID-containing proteins are involved in the regulation of a cell’s cycle, development, and differentiation [9,10]. In humans, the ARID family consists of 15 members, further categorized into 7 subfamilies based on their sequence and domain homology (Table 1) [5,11]. ARID1 and ARID2 are the core subunits of the BAF (BRGI/BRM associated factors) family of nucleosome remodeling complexes, whereas ARID3 are transcription factors [12]. Members of the ARID4 and ARID5 subfamilies serve as adaptor proteins that recruit transcriptional coregulators, such as the mSin3A-histone deacetylase (HDAC) complex and PHD Finger 2 (PHF2) histone demethylase [13,14,15]. JARID1 is involved in transcription regulation by the removal of histone H3K4 di-/tri-methylation marks [16], whereas JARID2 is a subunit of the Polycomb Repressive Complex 2 (PRC2) [17,18]. Herein, we focus on the ARID4 subfamily, particularly ARID4B, to summarize its prominent role in diverse biological processes, including development, differentiation, and human malignancies.

## 2. ARID4 Subfamily: ARID4A and ARID4B

### 2.1. Structural/Functional Domains of ARID4A and ARID4B

**Figure 1:**
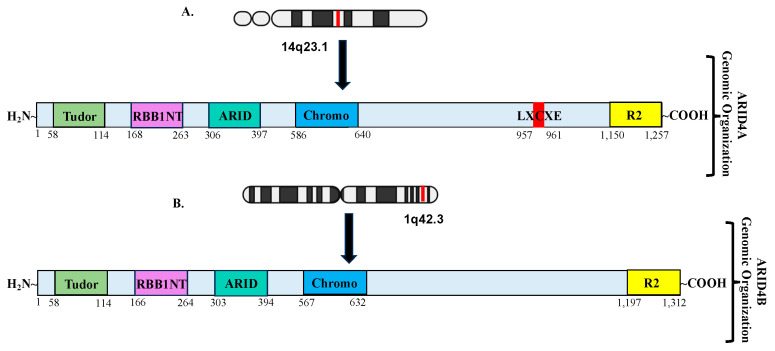
Genomic and domain organization of (**A**) ARID4A and (**B**) ARID4B. Relative positions of domains and motifs are represented by colored boxes. Human ARID4A protein encodes 1257 amino acids, and it shares an approximately 91% sequence identity with its mouse ortholog, while human ARID4B encodes 1312 amino acids that share a 95% similarity with mouse Arid4b with 1314 amino acids. Both ARID4A and ARID4B have one Tudor domain, one chromodomain, one ARID domain with a helix-turn-helix structure, and a repressor domain. Unlike ARID4B, ARID4A possesses an LXCXE sequence motif that facilitates binding to the retinoblastoma (RB) protein, thereby implicating it in RB-mediated transcriptional regulation.

**Figure 2:**
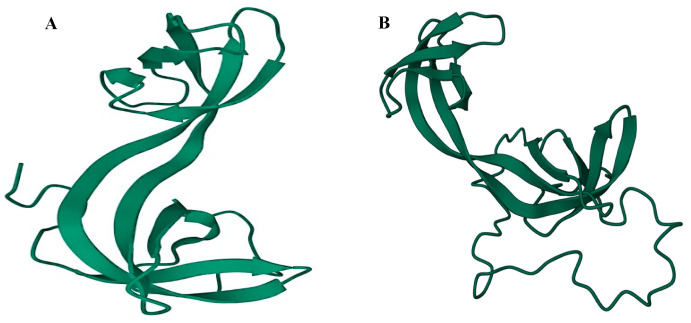
PDB solution structure of human (**A**) ARID4A and (**B**) ARID4B.

**Figure 3:**
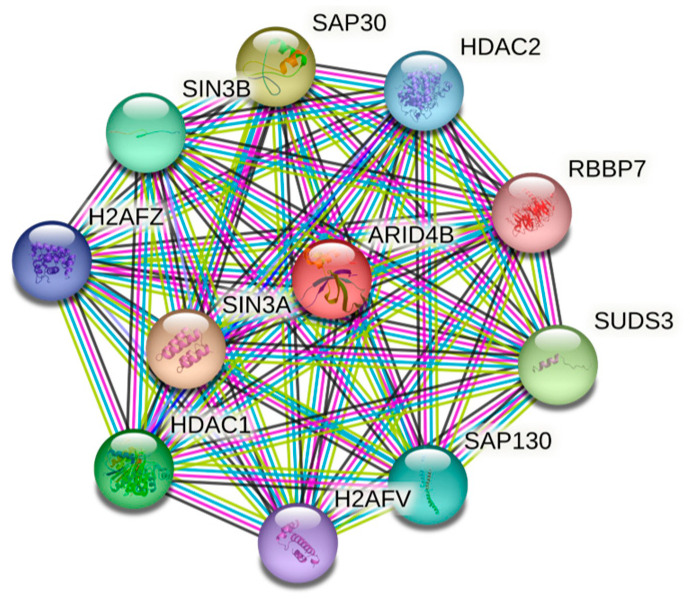
String database showing the interaction partners of ARID4B (ARID4B protein (human)–STRING interaction network).

ARID4A and ARID4B are the two members of the mammalian ARID4B subfamily, which are also known as retinoblastoma-binding protein 1 (RBBP1, RBP1) and RBBP1– like protein 1 (RBBP1L1), respectively [19,20]. These two proteins share an approximately 40–50% identity across the full length of the proteins and a 74% similarity within their ARID domains [5]. A LVCHE sequence, a conserved LXCXE motif that interacts with the retinoblastoma protein, was found in ARID4A but not in ARID4B. It has been reported that ARID4A functions as a transcriptional repressor of E2Fs [15,21,22]. Structurally, both ARID4A and ARID4B contain the ARID domain, the chromodomain, and the Tudor domain (Figure 1). In addition, they also contain two repressor domains, R1 and R2. The R1 domain appeared to overlap with the ARID domain, whereas the R2 is located at the C-terminus of the protein. The helix-turn-helix (HTH) motif is the most common DNA binding element. The HTH motif in the ARID domain confers potential DNA binding activity [5,22,23]. The chromodomain has been implicated in transcriptional regulation through its ability to bind to the methylated lysine residues on histones. Figure 2 describes the PDB solution structure of the human ARID4B Tudor domain. Though ARID4A and ARID4B share more than an 80% sequence similarity, the Tudor domain of ARID4B exhibited a significantly weaker DNA binding affinity. Interestingly, the structure determination and DNA titration analysis revealed the presence of Glu50 in ARID4B, instead of the Leu50 in ARID4A, and the former forms salt bridges with adjacent Leu residues at the DNA binding area, causing a decrease in the strength of its positive charge, ultimately resulting in a significantly low DNA binding affinity and increasing protein stability. The conserved RGR motif at the C-terminal extension of the ARID4B Tudor domain provides an additional DNA binding site. The difference in residues close to the DNA binding site and the presence of the RGR motif are highly conserved, making ARID4B functionally distinct from ARID4A [24]. In addition, both ARID4A and ARID4B were found to be components of the mSIN3–histone deacetylase suppressor complex [15,25], and Figure 3 explains all the interacting partners of ARID4B. While the expression of ARID4A is broadly detected, there is little or no expression of ARID4B in normal tissues, such as the stomach, pancreas, breast, spleen, colon, thymus, ovary, prostate, medulla, adrenal cortex, and PBMCs, but abundant expression in testis has been reported [26]. In stark contrast, an upregulation of ARID4B mRNA expression was detected in several cancers, including colon cancer, lung cancer, breast cancer, pancreatic cancer, and ovarian cancer, suggesting that ARID4B plays a vital role in tumorigenesis and could serve as a potential diagnostic marker for a range of human malignancies [26]. This review will explore the physiological and pathological roles of ARID4B in early embryogenesis and development, as well as cancer’s initiation, progression, and metastasis.

### 2.2. Functional Redundancy of Arid4a and Arid4b

Functional redundancy is a widespread mechanism in higher organisms where two or more genes perform the same function, and the inactivation of one has little or no impact on the biological phenotype [27,28,29]. Here, we highlight the functional redundancies existing between Arid4a and Arid4b.

A deficiency of Arid4a in mice follows a series of clinical conditions that are similar in humans, like defective hematopoiesis, chronic myelomonocytic leukemia (CMML)-like myelodysplastic/myeloproliferative disorder, and its final transformation into acute myeloid leukemia (AML) [30]. The mechanism by which Arid4a and Arid4b are involved in the acquisition of the leukemic phenotype was established utilizing wild-type, Arid4a^−/−^, and Arid4a^−/−^Arid4b^+/−^ mice. An absence of Arid4a in mice resulted in defective blood cell production in all hematopoietic lineages, with marked leukopenia and thrombocytopenia, in 2–5 months old mice. Once the animals were past 5 months of age, they exhibited monocytosis, severe anemia, and severe thrombocytopenia, with hemorrhage in the ovary of female mice ultimately resulting in female infertility. Splenomegaly and hepatomegaly are other important clinical conditions exhibited by Arid4a^−/−^ mice older than 5 months of age. Hematological malignancies are higher in Arid4a^−/−^ Arid4b^+/−^ mice when compared to Arid4a^−/−^, with a frequency of 83% and 12%, respectively [30]. Severe growth retardation, decreased body weight (30% reduction), and high mortality rates (25% of animals died before attaining 1 month of age) were observed in Arid4a^−/−^ Arid4b^+/−^ mice compared to Arid4a^−/−^ and wild-type mice. All these clinical situations, attributable to severe hematological malignancies seen in these animals, emphasize the functional redundancy of Arid4b.

The complete absence of Arid4a, combined with a haploinsufficiency of Arid4b (Arid4a^−/−^ Arid4b^+/−^), resulted in the progressive loss of male fertility in the Arid4a^−/−^Arid4b^+/−^ mouse model, highlighting an important point that Arid4a collaborates with Arid4b in the regulation of male fertility [31]. The study also underscored hypogonadism and seminal vesicle agenesis. Both Arid4a and Arid4b are expressed in the Sertoli cells of the seminiferous tubule, indicating its critical role in the process of spermatogenesis and providing an impermeable blood-testis barrier. Further evaluation of germ cell development in the Arid4a^−/−^Arid4b^+/−^ model indicated spermatogenic arrest, suggesting that both Arid4a and Arid4b play significant role in post-meiotic events. In addition, the increased permeability of the blood-testis barrier was also noticed in the seminiferous tubules of Arid4a^−/−^Arid4b^+/−^ mice. The key observation of the study conveys that the presence of two functional copies of Arid4a and/or Arid4b is sufficient to maintain normal fertility in Arid4a^−/−^ or Arid4a^+/−^Arid4b^+/−^ male mice [31].

### 2.3. ARID4B: An Orchestrator of Stem Cell Fate

Stem cells are unspecialized cells that are capable of differentiating extensively to any cell type (pluripotent) with the ability to self-renew. In higher eukaryotes, stem cells play critical role in the generation and maintenance of intercellular heterogeneity, which is linked to physiological tissue’s homeostasis [32]. Further, any numerical and functional disruptions in stem cell compartments could cause embryonic lethality, developmental deformities, and degenerative disorders that are related to aging and carcinogenesis [33]. Stem cells serve as a cardinal tool that provides a deeper insight into various physiological states like organogenesis and pathological situations like the development of cancer. Based on origin, stem cells are broadly classified into embryonic stem cells (ESCs), fetal and adult stem cells, and induced pluripotent stem cells (iPSCs). ESCs, derived from the inner cell mass of the blastocyst, can not only be maintained in an undifferentiated state in the culture media for a prolonged period but also differentiate to three primary germ layers [34,35]. The capability of ESCs to differentiate into any cell lineage has generated significant interest in stem cell research. A highly regulated interaction between the core pluripotency transcription factors and chromatin complexes is necessary to enable ESCs to execute the specific gene expression programs that determine cell fate [36,37,38]. ESCs undergo a reorganization of their chromatin architecture and genomic topology, and these alterations, in turn, impact the dynamics of lineage commitment [39,40,41,42,43,44]. The cell fate specification and the differentiation kinetics of ESCs during development are tightly regulated by the histone modification and chromatin architecture [45,46].

**Figure 4:**
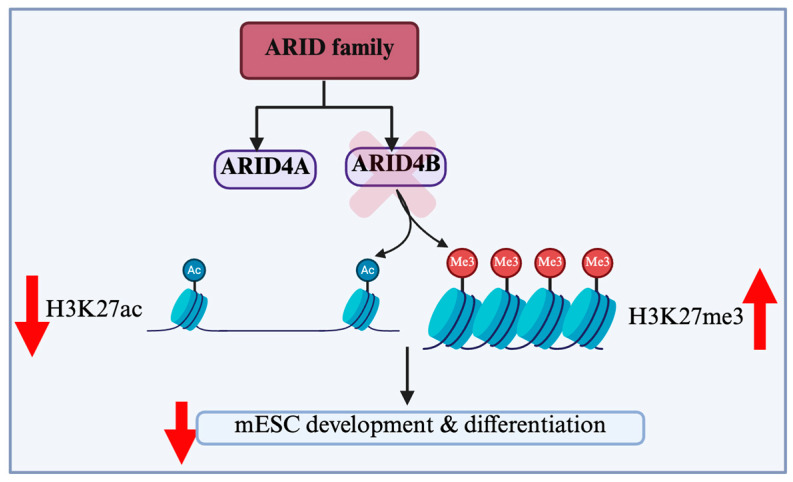
The ARID family in mESCs’ development and differentiation. ARID family proteins play a critical role in regulating gene expression during embryonic stem cells’ maintenance and differentiation. Their interactions with chromatin remodelers and transcription factors contribute to pluripotency and lineage commitment. A deficiency of ARID4B causes an increase in H3K27me3 levels and reduction in H3K27Aclevels in key developmental gene loci, resulting in aberrant gene expression during mESCs’ development and differentiation. ARID: AT-rich interaction domain, mESC: Mouse epithelial stem cells, H3K27me3: Trimethylation of lysine 27 on histone H3.

Mouse embryonic stem cells (mESCs) that are deficient in Arid4b are identical to wild-type mESCs with respect to the expression of pluripotency factors and their self-renewal capability [47]. However, a deficiency of Arid4b causes an increase in the H3K27me3 levels and concomitant reduction in H3K27Ac levels in key developmental gene loci (Figure 4). Thus, the knockdown or knockout of Arid4b results in aberrant gene expression during the exit of mESC pluripotency and meso/endodermal differentiation. Recently, Terzi et al. reported that ARID4B plays a significant role in mESCs’ development and differentiation [47] It was further revealed that Arid4b interacts with Tfap2c, whose consensus binding sequences are enriched in regions with an elevated H3K27Ac found in Arid4b-deficient mESCs [48]. These results suggest that Arid4b plays an important role in the execution of lineage-specific gene expression programs. In addition, during mESCs’ differentiation, Arid4b alters the dynamics between cell cycle and cell death [49]. During the onset of differentiation, Aridb4-deficient cells exhibit a lower proliferative capacity, indicating an important role of Arid4b in cell cycle rewiring. However, the exact mechanism behind this phenomenon is not completely understood.

This is in agreement with an earlier report, where whole-body *Arid4b* knockout in mice exhibited early embryonic lethality (E3.5-E7.5) [50]. Furthermore, a haplo-deficiency of *Arid4b* in adult mice promoted the development of acute myeloid leukemia in *Arid4a* null mice [30]. Interestingly, the expression of the *Foxp3* gene, which encodes a transcription factor critical for the development and function of regulatory T (Treg) cells, was downregulated in *Arid4a^−/−^ Arid4b^+/−^* mice.

Hematopoietic stem cells (HSCs) are oligopotent cells that can differentiate into a lymphoid and myeloid lineage [51]. A special microenvironmental niche in the bone marrow nurtures a pool of these HSCs to provide the mature blood cells required throughout our life, through the process of hematopoiesis [52,53]. HSCs and multipotency progenitor cells (MPPs) are the main components of the hematopoietic stem/progenitor cell (HSPC) pool. Long-term HSCs (LTHSCs) are capable of producing all types of hematopoietic cells and can give rise to MPP2, MPP3, and MPP4. In addition to LTHSCs, a transient repopulation of short-term HSCs (STHSCs) with the ability to proliferate and differentiate, with no or little self-renewing capacity, have also been identified. ST-HSCs are heterogenous and are often regarded as curative cell sources because of their ability to contribute to the repair of damaged tissues. In a vascular niche, they differentiate into myeloid- and lymphoid-lineage cells. However, they are capable of returning to dormancy after differentiation. [54,55,56,57]. The fundamental role of KIT signaling in the development of different cell types, like mast cells, erythrocytes and megakaryocytes, is well established by studies from spontaneous loss-of-function mutation in the murine white (W) spotting locus [58,59,60]. W locus mutation in mice correlated with self-renewal defects, whereas a gain of function mutation resulted in the augmented self-renewal capability of HSCs [61,62,63]. The KILG-mediated activation of the KIT receptor is essential for maintaining the homeostasis of the HSPC compartment through the tight balance existing between the self-renewing capacity of HSCs or differentiation to MPPs. A recent study revealed that the hematopoietic cell-specific knockout of Arid4b prevented hematopoiesis by blocking fetal HSC differentiation [64]. This study showed that HSCs that lack Arid4b aberrantly expressed KITLG and overexpressed KIT. Since a proper KILG-mediated activation of the KIT receptor is essential to maintain the homeostasis of the HSPC compartment, the dysregulated expression of KITLG and KIT in HSCs from Ardi4b knockout mice led to an aberrant activation of the KITLG/KIT-Src signaling pathway and failure of HSCs’ differentiation and hematopoiesis [64].

### 2.4. Spermatogenesis

Spermatogenesis is a tightly controlled process whereby spermatozoa are produced in the seminiferous tubules of the testis [65]. The spermatogenic germ cells are in contact with Sertoli cells, which provide nutrients and structural support for the developing germ cells. Therefore, Sertoli cells are important for spermatogenesis [65,66]. A Sertoli cell-specific Arid4b knockout mouse model (Arid4bSCKO) was generated. *Arid4bSCKO* male mice showed a significant reduction in testis size and complete infertility [67]. Further examination of the testes from *Arid4bSCKO* male mice revealed a loss of germ cells in the seminiferous tubules and a complete absence of mature sperm in the epididymis. A significant delay in spermatogenesis and an arrest at the spermatid stage were found in the testes of *Arid4bSCKO* mice. Mechanistically, it was revealed that ARID4B functions as a transcriptional coactivator for the androgen receptor and is required for the transcriptional activation of the AR target gene, reproductive homeobox5 (Rhox 5). Rhox 5 is expressed in Sertoli cells and plays an important role in spermatogenesis [67]. These results are not only consistent with the earlier findings that Arid4a ablation combined with Arid4b haploinsufficiency (Arid4a^−/−^Arid4b^+/−^) resulted in a loss of male fertility, but they also suggest the function of ARID4B in spermatogenesis is at least in part by regulating the expression of Rhox 5 [31].

Biologically, spermatogenesis is a developmental process where spermatogonial stem cells (SSCs) undergo a series of differentiation processes, ultimately giving rise to spermatozoa. During the neonatal development, the primary SSCs are formed from the precursor gonocytes. The SSCs serve as a foundational reservoir of spermatozoa throughout adulthood [68]. The temporospatial establishment of a niche by the Sertoli cells is critical for the gonocyte–SSC transformation. However, the factors that regulate the establishment of this niche remain largely unknown.

Interestingly, ARID4B not only regulated spermatogenesis but also was reported to regulate the establishment of a niche important for gonocyte–SSC transition [69]. During gonocyte–SSC transition, an abnormal detachment of Sertoli cells from the basement membrane of seminiferous tubules was observed in Arid4bSCKO mice. The failure in establishing the niche led to an abnormal gonocyte distribution that ultimately resulted in apoptosis. Gene expression profiling identified a panel of genes that are important in the stem cell niche function, including anti-Mullerian hormone, Glial cell-line-derived neurotrophic factor, gap junction protein alpha-1, inhibin alpha and beta, the KIT ligand, and cytochrome P450 family 26 subfamily b polypeptide 1, whose expressions were affected in *Arid4bSCKO* mice. [69] In agreement with these findings, a Transmission Ratio Distortion (TRD) study identified a panel of genes, including ARID4B, GSK3B, NSMCE1, AK7, ZC3H13, BDKRB2, PALB2, VRK1, and NID1, to be candidate genes involved in spermatogenesis in swine [70]. Taken together, these results suggest that ARID4B is a master regulator of spermatogenesis, and the function of ARID4B in spermatogenesis is conserved across different species. Table 2 summarizes the involvement of ARID4B in various physiological processes. 

## 3. The Role of ARID4B in Cancer

### 3.1. Prostate Cancer

**Figure 5:**
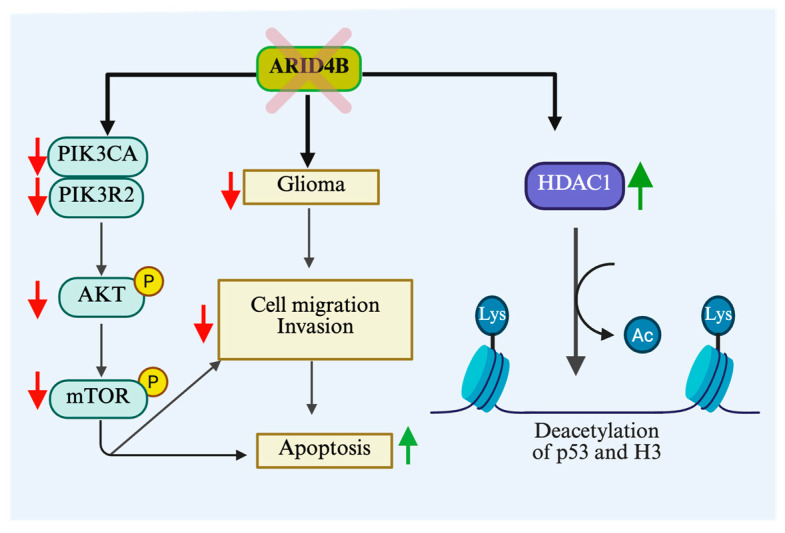
Downregulation of ARID4B leads to the suppression of key oncogenic signaling molecules, including PIK3CA, PIK3R2, AKT, and mTOR, thereby reducing cell proliferation, invasion, and migration while promoting apoptosis. In glioma, ARID4B downregulation decreases tumors’ progression and metastatic potential by impairing PI3K/AKT/mTOR pathway activation, ultimately enhancing apoptotic cell death. Additionally, ARID4B contributes to epigenetic regulation by facilitating the deacetylation of p53 and histone H3 (H3K9/H3K27), leading to chromatin remodeling and the transcriptional repression of tumor suppressor genes, further promoting apoptosis and inhibiting tumorigenesis. mTOR: Mammalian target of rapamycin, PIK3R2: Phosphoinositide-3-Kinase Regulatory Subunit 2, PIK3CA: Phosphatidylinositol-4,5-bisphosphate 3-kinase catalytic subunit alpha, Lys: Lysine, HDAC1: Histone deacetylase 1.

Prostate cancer is the second most common malignancy and a leading cause of cancer-related death in men in Western countries. Epidemiological evidence supports a strong genetic contribution to prostate cancer susceptibility [71,72]. Men with inherited variants in particular genes, such as BRACA1, BRACA2, and HOXB13, have a higher risk of developing prostate cancer in their lifetimes [73]. In addition, men with BRACA2 or HOXB13 gene variants may have a higher risk of developing life-threatening forms of prostate cancer [74,75,76]. Genome-wide association studies (GWASs) revealed the involvement of more than 150 single nucleotide variants (SNVs) in the risk of prostate cancer and its progression [77]. The greatest prostate cancer risk is associated with variations in two DNA damage repair genes, BRACA2 and HOXB13. However, a panel of genes that include BRACA1, MSH2, MSH6, CHEK2, PALB2, ATM, RAD51D, PMS2, and NBS1 were reported to be involved in the early onset of prostate cancer [78,79,80,81]. Fusions of TMPRSS2 with the ETS family of genes, such as ERG and ET1, the amplification of the oncogene MYC, deletion or mutations of tumor suppressors TP53 and PTEN, and amplification of the androgen receptor (AR) are other key genetic alterations found in different stages in prostate cancer [80,82,83,84,85,86]. Recently, a study using prostate-specific Arid4b knockout mouse models showed that ARID4B is essential for the initiation and progression of PTEN-deficient prostate cancer, suggesting the synthetic essentiality of ARID4B in prostate cancer carries PTEN deletion (Figure 5) [87]. The study demonstrated that ARID4B regulates the PTEN-AKT signaling pathway by regulating the expression of PIK3CA and PIK3R2 through a modulation of the chromatin structure on PIK3CA and PIK3R2 promoters. PTEN deficiency results in the activation of the PI3K-AKT pathway and prostate tumorigenesis. Therefore, the ablation of Arid4b inhibited the PI3K-AKT activation and prostate tumorigenesis driven by a PTEN deficiency. These results also suggest that ARID4B is a potential therapeutic target for PTEN-deficient prostate cancer [87]. In prostate cancer, miR-30d binds to the 3′ UTR of ARID4A and ARID4B, resulting in the downregulation of these molecules and enhanced cancer progression. In prostate cancer patients, PSA failure, a high Gleason score, and a shorter BCR-free survival were linked with the downregulated expression of ARID4B. Altogether, the co-downregulation of ARID4A and ARID4B could serve as a prognostic biomarker in prostate cancer patients [88].

### 3.2. Breast Cancer

Breast cancer is the most common cancer among women around the world [89]. It was estimated that one in every eight women will develop breast cancer in their lifetimes [90,91]. The potential involvement of ARID4B in breast cancer was first suggested by the physical interaction between ARID4B and BRMS1 (breast cancer metastasis suppressor) [92]. Recently, increased ARID4B expression was reported to correlate with unfavorable clinical outcomes in patients with breast cancer [93]. Allelic variation and the differential expression of ARID4B attributed to mammary tumor growth and metastasis in ER+ breast cancer [92]. The knockdown of *Arid4b* significantly reduced pulmonary metastasis in mouse models. Concomitant with the loss of metastatic efficiency in *Arid4b* knockdown cells, the downregulation of the Tpx2 gene network, which is involved in regulating cell cycle and mitotic spindle biology, was identified. By profiling miRNA expression using high- and low-metastatic mouse strains, the study further revealed a correlation of miR-290 with mammary metastatic burden. Bioinformatics analysis identified Arid4b as the top target of miR-290, suggesting that miR-290 might suppress breast cancer’s progression by targeting Arid4b [94].

### 3.3. Colorectal Carcinoma

**Figure 6:**
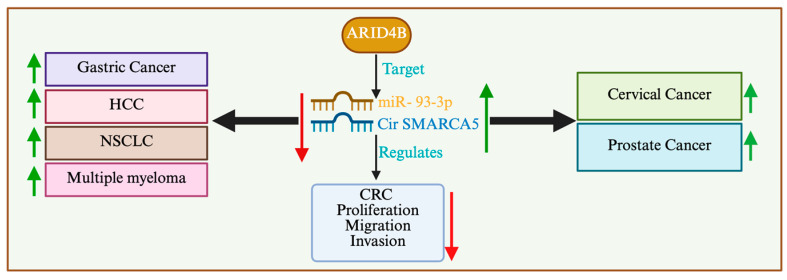
ARID4B regulates MiR-93-3p through chromatin remodeling, influencing cancer progression across multiple malignancies, including colorectal, cervical, prostate, gastric, lung (NSCLC), hepatocellular carcinoma (HCC), and multiple myeloma. MiR-93, upregulated by ARID4B, suppresses tumor suppressors such as PTEN, p21 (CDKN1A), LATS2, and FOXO1, leading to enhanced cell proliferation, invasion, metastasis, and chemoresistance. In CRC and gastric cancer, MiR-93 promotes EMT and angiogenesis by targeting E-cadherin and VEGF, while in prostate cancer, it enhances androgen receptor signaling and castration resistance. In NSCLC and HCC, MiR-93 suppresses RB1 and p53, accelerating tumor growth. In multiple myeloma, it facilitates immune evasion and therapy resistance by activating NF-κB. Targeting the ARID4B-MiR-93 axis presents a potential therapeutic strategy to restore tumor suppressor pathways and inhibit metastatic progression. MiR-93-3p: microRNA 93-3p, circ-SMARCA5: circular RNA SMARCA5, NSCLC: non-small cell lung cancer.

Colorectal carcinoma (CRC) remains a global health issue, despite advances in the medical sciences reducing the incidence and mortality rates of this cancer in many countries [95]. The initiation and progression of CRC can be attributed to multiple genetic and epigenetic alterations [96]. SMARCA5 (SWI/SNF-related, matrix-associated, actin-dependent regulator of chromatin, subfamily a, member 5) is a member of the ISWI family of proteins, which contain helicase and ATPase activities known to regulate gene transcription by altering the chromatin structure. The role of SMARCA5 in cancer appears to be tissue type-specific, as the expression of SMARCA5 is upregulated in cervical and prostate cancers, whereas downregulation is reported in multiple myeloma, HCC, gastric cancer, intrahepatic cholangiocarcinoma, and non-small cell lung cancer (Figure 6) [97,98,99,100]. Recently, it was reported that circ-SMARCA5 expression was dramatically decreased in CRC cell lines and tissues [101]. It was further revealed that circ-SMARCA5 overexpression inhibited CRC cells’ proliferation, migration, and invasion by targeting miR-93-3p. Interestingly, ARID4B was predicted as the target of miR-93-3p and circ-SMARCA5-upregulated ARID4B expression via miR-93–3p. In addition, it was recently reported that the expression of miR-519b-3p correlated with the responsiveness to preoperative chemoradiotherapy (pCRT) in patients with locally advanced rectal cancer (LARC) [102]. The study further showed that miR-519b-3p directly binds to the 3′ UTR of ARID4B mRNA, whose expression is inversely correlated with miR-519b-3p expression, suggesting that miR-519b-3p promotes responsiveness to pCRT by suppressing ARID4B expression. These results suggest that the miR-519b-3p/ARID4B axis may serve as a predictive marker for the response to CRT in LARC.

### 3.4. Hepatocellular Carcinoma

Hepatocellular carcinoma accounts for 75–85% of primary liver cancer, and it is the fourth leading cause of cancer-related death worldwide [103,104]. Limitations in treatment modalities and high resistance rates to conventional chemotherapeutic drugs contribute to the poor prognosis of HCC, with a 5-year survival rate at 18%. Comprehensive analysis using the TCGA datasets showed that the expressions of several members of the ARID family, including ARID4B, were elevated in HCC specimens compared to non-tumor specimens [105]. In addition, it was reported that ARID4B acts as an oncogene in HCC, and increased ARID4B expression is correlated with vascular invasion, tumor node metastasis, tumor burden, high Edmondson–Steiner grades, and the poor prognosis of patients [106].

### 3.5. Glioblastoma

Glioblastoma is the most aggressive and common form of brain cancer. MiRNA-based therapies and epigenetic drugs have also been proposed for the treatment of glioblastoma [107]. Overexpression of ARID4B has been detected in a majority of primary brain tumors, and the expression of ARID4B correlated with higher grades of glioma [108]. The knockdown of ARID4B in the glioma cell lines LN229 and GMB8401 was shown to significantly reduce glioma cell migration and invasion, accompanied with increased apoptosis. In line with its function in prostate cancer, the knockdown of ARID4B also resulted in a reduced expression of p-AKT and p-mTOR levels. In addition, the knockdown of ARID4B induced G1 cell cycle arrest, resulting in decreased glioma cell proliferation via inhibition of the PI3K/AKT pathway and downregulation of cyclin D1 expression. Interestingly, the authors reported an upregulated expression of HDAC1 upon ARID4B knockdown, which, in turn, led to the deacetylation of p53 (a reduction in the level of acetyl-p53) and H3 (reduced acetyl-H3). However, the effects of P53 and H3 deacetylation on glioma cells (as shown in Figure 5) remain unknown [109]. Table 3 and Figure 6 summarize the involvement of ARID4B in various cancer types.

## 4. Conclusions and Future Perspectives

**Figure 7:**
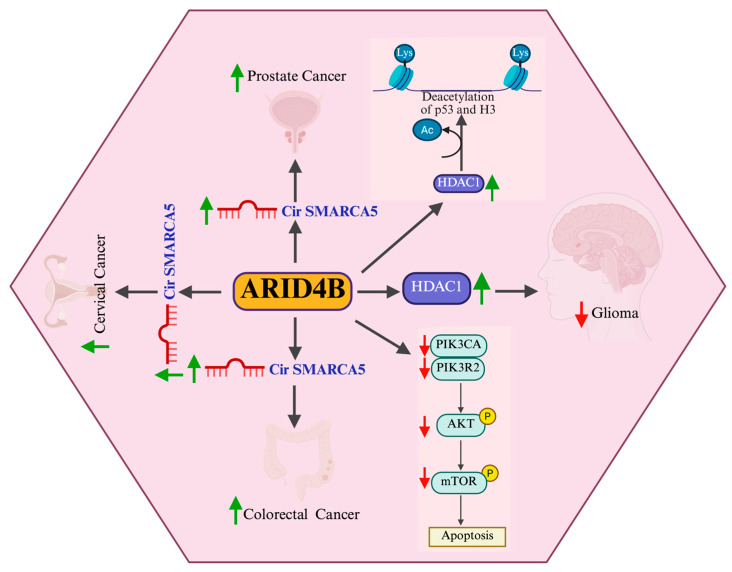
Schematic showing the up- and downregulation of ARID4B in various physiological and pathological states. ARID4B Upregulation: ARID4B upregulation is associated with circSMARCA5, which contributes to prostate, cervical, and colorectal cancer’s progression. It influences the PI3K/AKT/mTOR pathway, leading to PIK3CA and PIK3R2’s downregulation, reducing PIP2-to-PIP3 conversion, and inhibiting AKT phosphorylation which results in mTOR inhibition, promoting apoptosis while reducing the proliferation, migration, and invasion of cancer cells. ARID4B Downregulation: ARID4B downregulation leads to increased HDAC1 activity, causing the deacetylation of p53 and histone H3, affecting gene regulation. It promotes H3K27me3 histone methylation, leading to reduced mESC (mouse embryonic stem cell) development and differentiation. The loss of ARID4B activity in gliomas results in increased cell migration and invasion, while also altering apoptosis mechanisms. circSMARCA5—Circular RNA SMARCA5 (actin-dependent regulator of chromatin subfamily A member 5), PI3K—Phosphoinositide 3-Kinase, AKT—Protein Kinase B (PKB), mTOR—Mechanistic Target of Rapamycin, PIP2—Phosphatidylinositol 4,5-bisphosphate, PIP3—Phosphatidylinositol 3,4,5-trisphosphate, HDAC1—Histone Deacetylase 1, p53—Tumor Protein p53, H3K27me3—Trimethylation of Histone H3 at Lysine 27.

Significant progress has been made in understanding the role of epigenetic modifications in human health and disease. This review highlights the multifaceted functions of ARID4B in key biological processes, including embryogenesis, spermatogenesis, and the development of cancer (Figure 7). Acting as both an oncogene and a tumor suppressor, ARID4B influences tumors’ progression and metastasis through its context-dependent regulation of cellular signaling pathways. Insights gained from various ARID4B knockout murine models (Arid4bPC^−/−^, Arid4a^−/−^, Arid4b^+/−^, Arid4bHC^−/−^, Arid4bSCKO) have significantly advanced our understanding of its mechanistic roles, paving the way for evaluating ARID4B as a potential therapeutic target. Future studies employing advanced technologies and comprehensive in vivo and in vitro models will further clarify its contributions to other cancer types. Collectively, the evidence supports ARID4B as a promising biomarker and therapeutic target, offering innovative avenues for the diagnosis, prognosis, and treatment of diverse human malignancies.

## Figures and Tables

**Table 1:** Genome distribution of Arid gene family in human and mouse.

Human		Mouse	
*ARID1A*	Chromosome 1p36.11	*Arid1a*	Chromosome 4qD2.3
*ARID1B*	Chromosome 6q25.3	*Arid1b*	Chromosome 17qA1
*ARID2*	Chromosome 12q12	*Arid2*	Chromosome 15qF1
*ARID3A*	Chromosome 19p13.3	*Arid3a*	Chromosome 10qC1
*ARID3B*	Chromosome 15q24.1	*Arid3b*	Chromosome 9qB
*ARID3C*	Chromosome 9p13.3	*Arid3c*	Chromosome 10qC1
*ARID4A*	Chromosome 14q23.1	*Arid4a*	Chromosome 12qC2
*ARID4B*	Chromosome 1q42.3	*Arid4b*	Chromosome 13qA1
*ARID5A*	Chromosome 2q11.2	*Arid5a*	Chromosome 1qB
*ARID5B*	Chromosome 10q21.2	*Arid5b*	Chromosome 10qB5.2
*JARID1A*	Chromosome 12p13.33	*Jarid1a*	Chromosome 6qF1
*JARID1B*	Chromosome 1q32.1	*Jarid1b*	Chromosome 1qE4
*JARID2*	Chromosome 6p22.3	*Jarid2*	Chromosome 13qA5
*JARID1C*	Chromosome Xp11.22	*Jarid1c*	Chromosome XqF3
*JARID1D*	Chromosome Yq11.223	*Jarid1d*	Chromosome YqA1

**Table 2:** Summary of the involvement of Arid4b in stem cell fate.

Physiological State	Model System	Associated Cellular Mechanisms	Reference
ESC renewal	*In vitro*	Arid4b regulates transcriptional networks critical for maintaining pluripotency and self-renewal of ESCs	[30,31,51]
Impaired hematopoietic stem cell function	*In vivo*, *In vitro*, *In silico*	Arid4b maintains HSPC homeostasis by regulating self-renewal, differentiation, and lineage commitment via the KITLG/KIT-Src signaling axis	[64]
Embryonic germ layer differentiation	*In vitro*	Arid4b binds to the transcription factor Tfap2c, regulating the differentiation of endoderm and mesoderm	[48]
Cell cycle regulation and apoptosis during embryonic development	*In vitro*	Arid4b altered cell cycle and cell death in a Caspase 3-dependent manner	[49]
Hormonal regulation of male germ cell development	*In vivo*, *In vitro*	Arid4b coactivates the androgen receptor (AR), which modulates AR-dependent transcription essential for the development and maturation of male germ cells	[67]
Stem cell niche establishment during spermatogenesis	*In vivo*	Arid4b regulates the signaling network required to establish a niche in the gonocyte–spermatogonial stem cell transition	[69]

**Table 3:** Summary of the involvement of Arid4b in multiple human malignancies.

Cancer Type	Model System	Oncogene/Tumor Suppressor	Associated Cellular Functions	Reference
Prostate cancer	*In vivo*, *In vitro*	Oncogene	Arid4b regulates PIK3CA and PIK3R2, leading to PTEN downregulation and AKT pathway activation, thereby promoting prostate cancer cell survival and proliferation.	[87]
Prostate cancer	*In vivo*, *In vitro*	Tumor suppressor	Arid4b expression is negatively regulated by miRNA-30d, leading to reduced tumor suppressor activity	[88]
Breast cancer	*In vivo*, *In vitro*	Oncogene	Arid4b mediates estrogen receptor signaling through miRNA-290 regulation, promoting cancer cell proliferation and survival	[94]
Breast cancer	*In vivo*, *In vitro*	Oncogene	Arid4b inhibits pulmonary metastasis by interacting with BRMS1, indicating its complex role in regulating metastatic potential	[92]
Hepatocellular carcinoma	*In vivo*	Oncogene	Arid4b overexpression suggests a potential role in liver tumor development or progression	[106]
Glioblastoma	*In vitro*, *In silico*	Oncogene	Knockdown of Arid4b favors apoptosis through PI3K/AKT pathway	[102]
Glioblastoma	*In vivo*, *In vitro*	Oncogene	Enhanced expression of Arid4b in primary brain tumor highly correlated with WHO grades	[108]
Acute myeloid leukemia	*In vivo*	Tumor suppressor	Haplo-deficiency of *Arid4b* in adult mice enhanced onset of acute myeloid leukemia in Arid4a null mice	[30]
Colorectal cancer	*In vivo*, *In vitro*	Tumor suppressor	miRNA-mediated activation of cSMARCA5/miR-39a-3p/ARID4B pathway enhances Arid4b expression and inhibits tumorigenesis	[101]
Colorectal cancer	*In vivo*, *In vitro*	Oncogene	Enhanced cellular responsiveness to chemoradiation through binding of miR-519b-3p to Arid4b	[102]

## Data Availability

No new data were created or analyzed in this study.

## Abbreviations

The following abbreviations are used in this manuscript:

AKT	Protein Kinase B
ARID	AT rich interaction domains
circ-SMARCA5	Circular RNA SMARCA5
H3K27me3	Trimethylation of Histone H3 at Lysine 27
HDAC1	Histone Deacetylase 1
Lys	Lysine
mTOR	Mammalian target of rapamycin
NSCLC	Non-small cell lung cancer
p53	Tumor Protein p53
PI3K	Phosphoinositide 3-Kinase
PIK3CA	Phosphatidylinositol-4,5-bisphosphate 3-kinase catalytic subunit alpha
PIK3R2	Phosphoinositide-3-Kinase Regulatory Subunit 2
PIP2	Phosphatidylinositol 4,5-bisphosphate
PIP3	Phosphatidyl-inositol 3,4,5-trisphosphate
